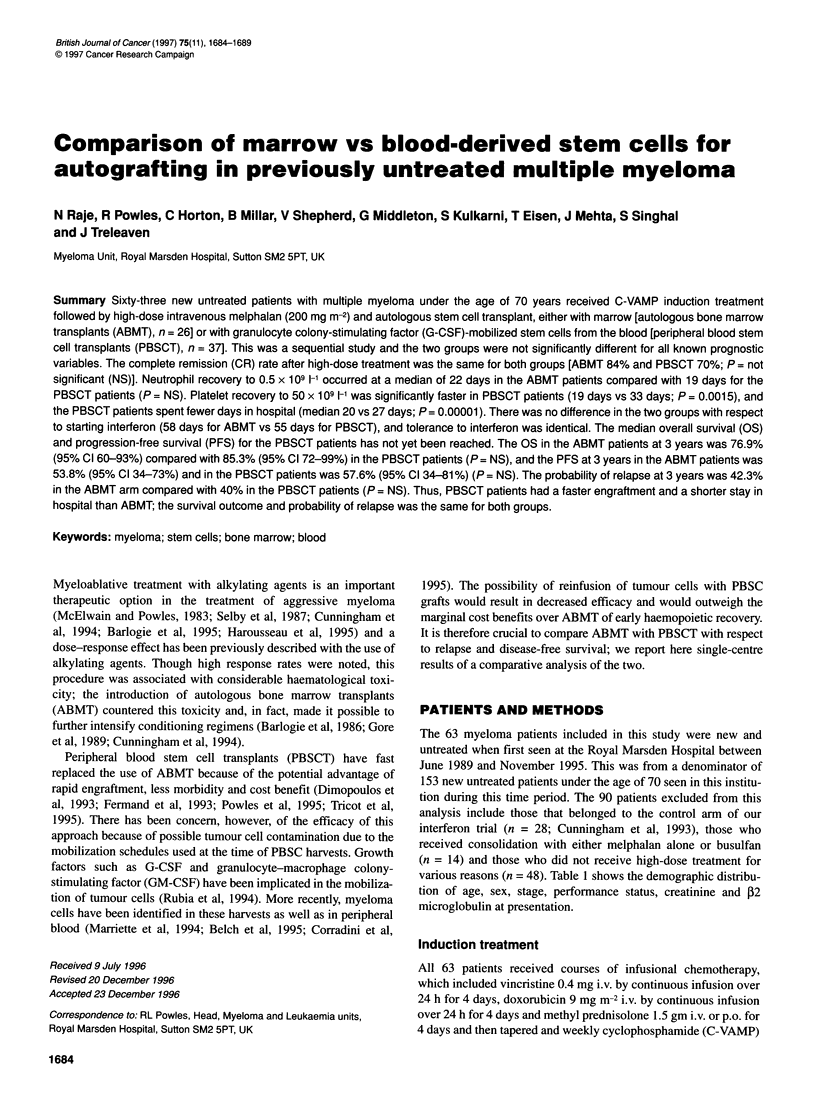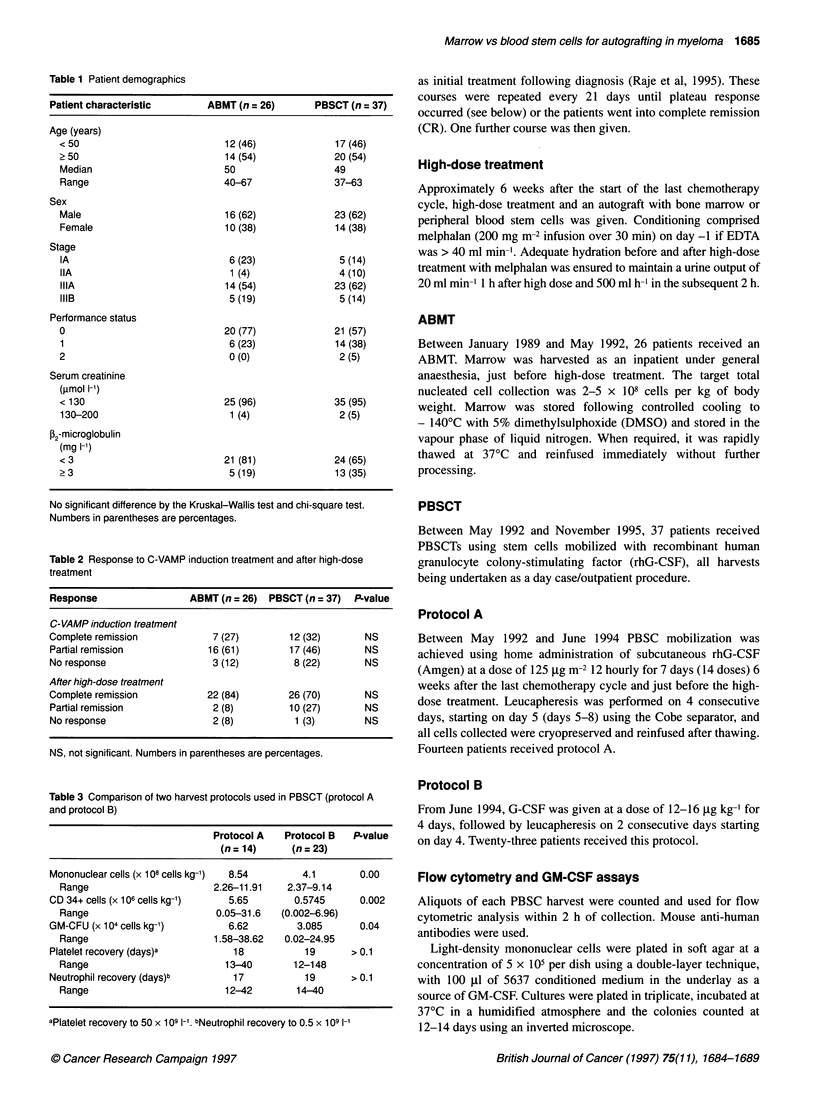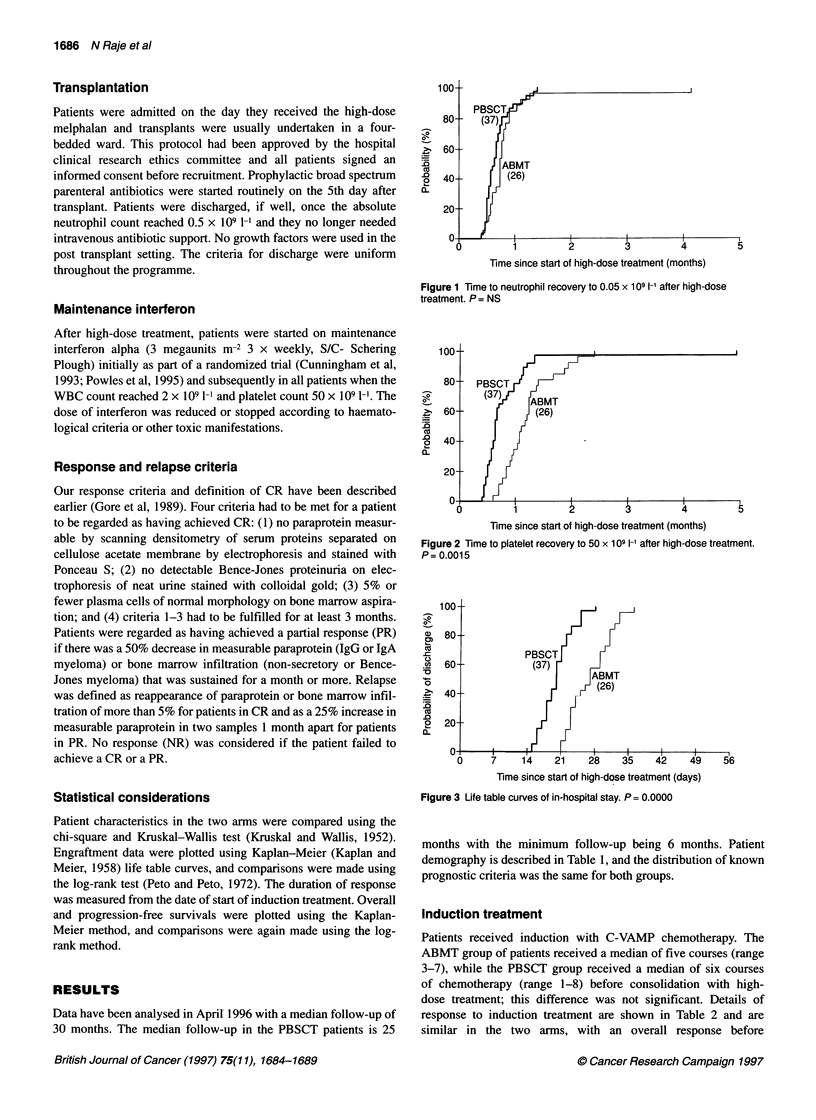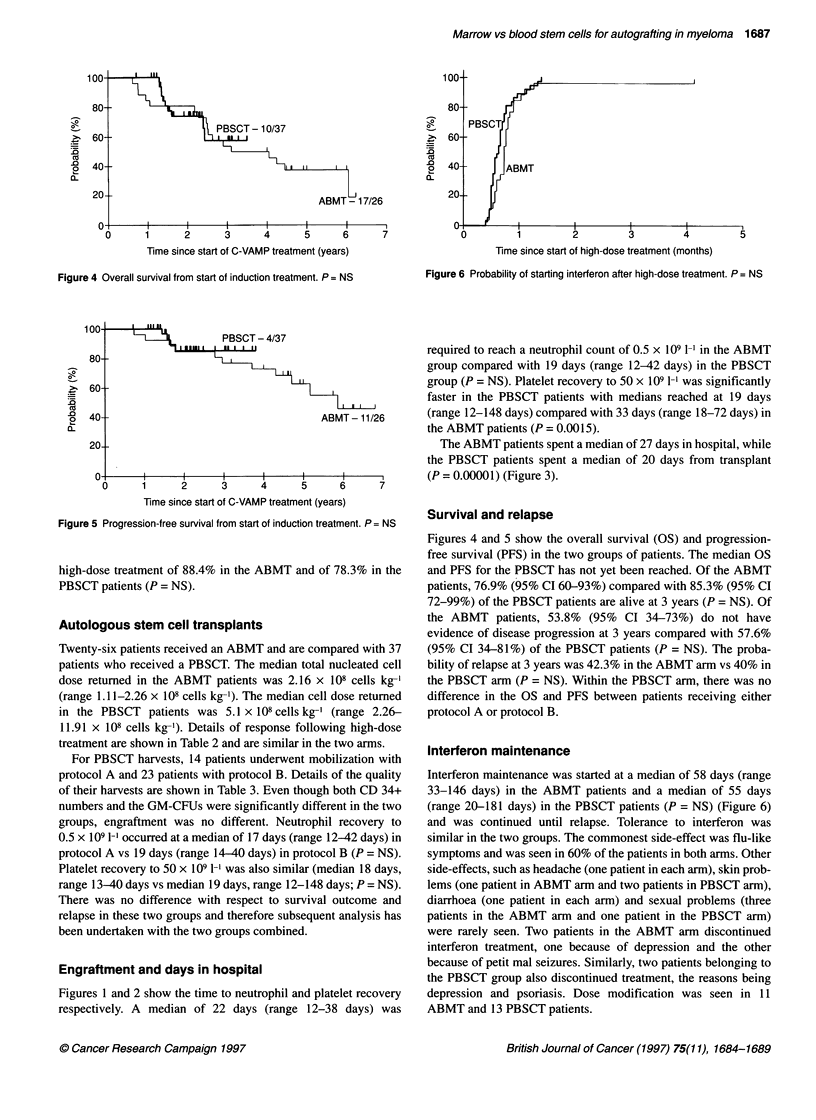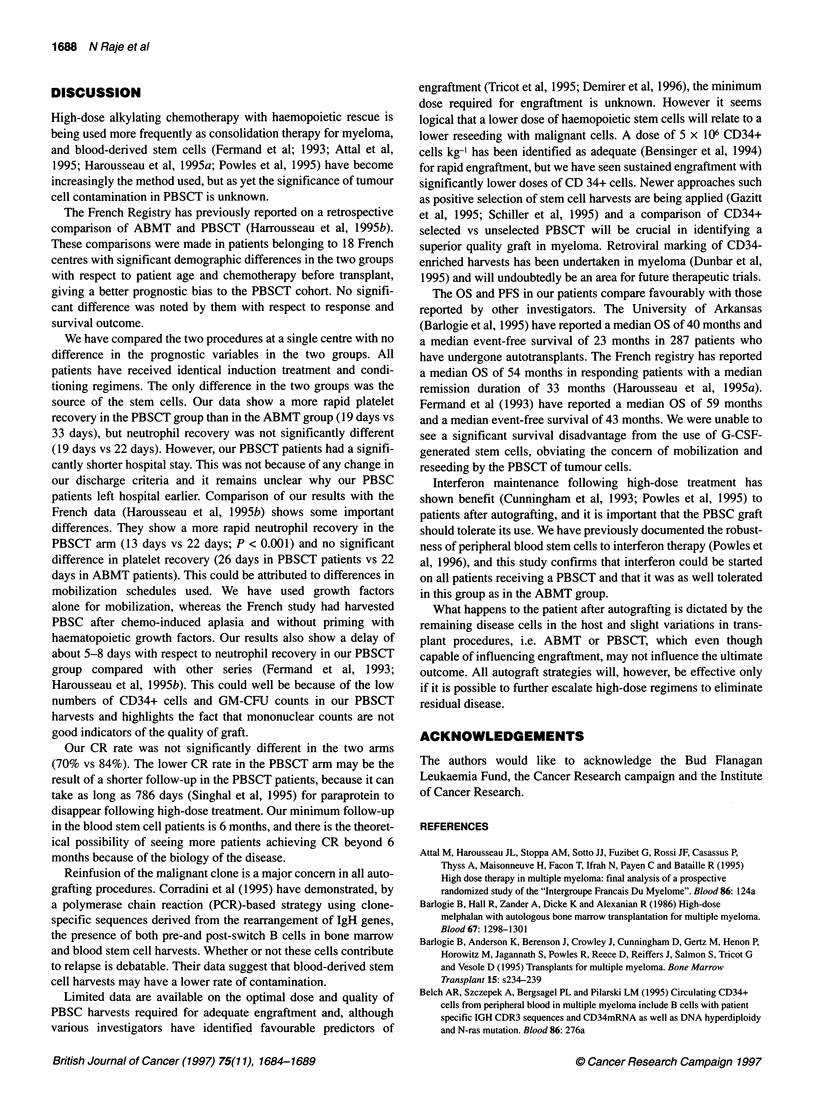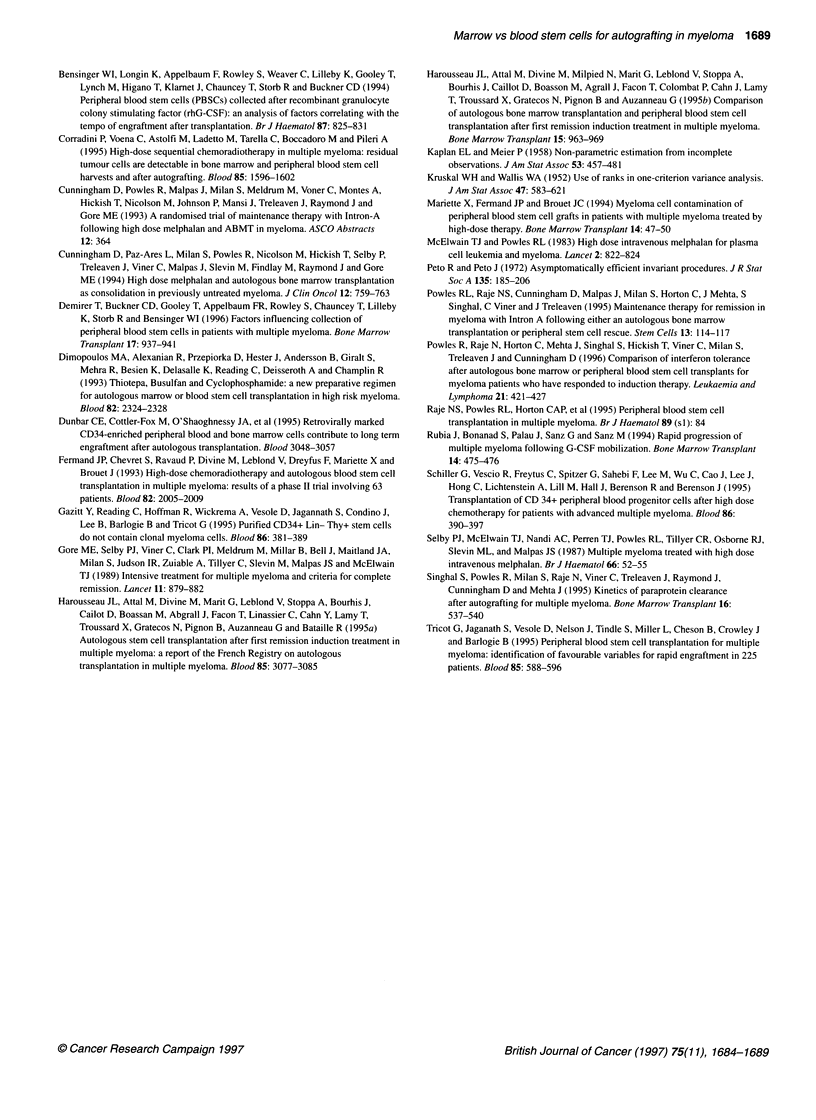# Comparison of marrow vs blood-derived stem cells for autografting in previously untreated multiple myeloma.

**DOI:** 10.1038/bjc.1997.286

**Published:** 1997

**Authors:** N. Raje, R. Powles, C. Horton, B. Millar, V. Shepherd, G. Middleton, S. Kulkarni, T. Eisen, J. Mehta, S. Singhal, J. Treleaven

**Affiliations:** Myeloma Unit, Royal Marsden Hospital, Sutton, UK.

## Abstract

Sixty-three new untreated patients with multiple myeloma under the age of 70 years received C-VAMP induction treatment followed by high-dose intravenous melphalan (200 mg m(-2)) and autologous stem cell transplant, either with marrow [autologous bone marrow transplants (ABMT), n = 26] or with granulocyte colony-stimulating factor (G-CSF)-mobilized stem cells from the blood [peripheral blood stem cell transplants (PBSCT), n = 37]. This was a sequential study and the two groups were not significantly different for all known prognostic variables. The complete remission (CR) rate after high-dose treatment was the same for both groups [ABMT 84% and PBSCT 70%; P = not significant (NS)]. Neutrophil recovery to 0.5 x 10(9) l(-1) occurred at a median of 22 days in the ABMT patients compared with 19 days for the PBSCT patients (P = NS). Platelet recovery to 50 x 10(9) l(-1) was significantly faster in PBSCT patients (19 days vs 33 days; P = 0.0015), and the PBSCT patients spent fewer days in hospital (median 20 vs 27 days; P = 0.00001). There was no difference in the two groups with respect to starting interferon (58 days for ABMT vs 55 days for PBSCT), and tolerance to interferon was identical. The median overall survival (OS) and progression-free survival (PFS) for the PBSCT patients has not yet been reached. The OS in the ABMT patients at 3 years was 76.9% (95% CI 60-93%) compared with 85.3% (95% CI 72-99%) in the PBSCT patients (P = NS), and the PFS at 3 years in the ABMT patients was 53.8% (95% CI 34-73%) and in the PBSCT patients was 57.6% (95% CI 34-81%) (P = NS). The probability of relapse at 3 years was 42.3% in the ABMT arm compared with 40% in the PBSCT patients (P = NS). Thus, PBSCT patients had a faster engraftment and a shorter stay in hospital than ABMT; the survival outcome and probability of relapse was the same for both groups.


British Journal of Cancer (1997) 75(11), 1684–1689
© 1997 Cancer Research Campaign

### Comparison of marrow vs blood-derived stem cells for autografting in previously untreated multiple myeloma

**N Raje, R Powles, C Horton, B Millar, V Shepherd, G Middleton, S Kulkarni, T Eisen, J Mehta, S Singhal and J Treleaven**

Myeloma Unit, Royal Marsden Hospital, Sutton SM2 5PT, UK

**Summary** Sixty-three new untreated patients with multiple myeloma under the age of 70 years received C-VAMP induction treatment followed by high-dose intravenous melphalan (200 mg $m^{-2}$) and autologous stem cell transplant, either with marrow [autologous bone marrow transplants (ABMT), $n$ = 26] or with granulocyte colony-stimulating factor (G-CSF)-mobilized stem cells from the blood [peripheral blood stem cell transplants (PBSCT), $n$ = 37]. This was a sequential study and the two groups were not significantly different for all known prognostic variables. The complete remission (CR) rate after high-dose treatment was the same for both groups [ABMT 84% and PBSCT 70%; $P$ = not significant (NS)]. Neutrophil recovery to $0.5 \times 10^9$ $l^{-1}$ occurred at a median of 22 days in the ABMT patients compared with 19 days for the PBSCT patients ($P$ = NS). Platelet recovery to $50 \times 10^9$ $l^{-1}$ was significantly faster in PBSCT patients (19 days vs 33 days; $P$ = 0.0015), and the PBSCT patients spent fewer days in hospital (median 20 vs 27 days; $P$ = 0.00001). There was no difference in the two groups with respect to starting interferon (58 days for ABMT vs 55 days for PBSCT), and tolerance to interferon was identical. The median overall survival (OS) and progression-free survival (PFS) for the PBSCT patients has not yet been reached. The OS in the ABMT patients at 3 years was 76.9% (95% CI 60–93%) compared with 85.3% (95% CI 72–99%) in the PBSCT patients ($P$ = NS), and the PFS at 3 years in the ABMT patients was 53.8% (95% CI 34–73%) and in the PBSCT patients was 57.6% (95% CI 34–81%) ($P$ = NS). The probability of relapse at 3 years was 42.3% in the ABMT arm compared with 40% in the PBSCT patients ($P$ = NS). Thus, PBSCT patients had a faster engraftment and a shorter stay in hospital than ABMT; the survival outcome and probability of relapse was the same for both groups.

**Keywords:** myeloma; stem cells; bone marrow; blood

Myeloablative treatment with alkylating agents is an important therapeutic option in the treatment of aggressive myeloma (McElwain and Powles, 1983; Selby et al, 1987; Cunningham et al, 1994; Barlogie et al, 1995; Harousseau et al, 1995) and a dose–response effect has been previously described with the use of alkylating agents. Though high response rates were noted, this procedure was associated with considerable haematological toxicity; the introduction of autologous bone marrow transplants (ABMT) countered this toxicity and, in fact, made it possible to further intensify conditioning regimens (Barlogie et al, 1986; Gore et al, 1989; Cunningham et al, 1994).

Peripheral blood stem cell transplants (PBSCT) have fast replaced the use of ABMT because of the potential advantage of rapid engraftment, less morbidity and cost benefit (Dimopoulos et al, 1993; Fermand et al, 1993; Powles et al, 1995; Tricot et al, 1995). There has been concern, however, of the efficacy of this approach because of possible tumour cell contamination due to the mobilization schedules used at the time of PBSC harvests. Growth factors such as G-CSF and granulocyte–macrophage colony-stimulating factor (GM-CSF) have been implicated in the mobilization of tumour cells (Rubia et al, 1994). More recently, myeloma cells have been identified in these harvests as well as in peripheral blood (Marriette et al, 1994; Belch et al, 1995; Corradini et al, 1995). The possibility of reinfusion of tumour cells with PBSC grafts would result in decreased efficacy and would outweigh the marginal cost benefits over ABMT of early haemopoietic recovery. It is therefore crucial to compare ABMT with PBSCT with respect to relapse and disease-free survival; we report here single-centre results of a comparative analysis of the two.

*Received 9 July 1996*
*Revised 20 December 1996*
*Accepted 23 December 1996*

*Correspondence to:* RL Powles, Head, Myeloma and Leukaemia units, Royal Marsden Hospital, Sutton SM2 5PT, UK

### PATIENTS AND METHODS

The 63 myeloma patients included in this study were new and untreated when first seen at the Royal Marsden Hospital between June 1989 and November 1995. This was from a denominator of 153 new untreated patients under the age of 70 seen in this institution during this time period. The 90 patients excluded from this analysis include those that belonged to the control arm of our interferon trial ($n$ = 28; Cunningham et al, 1993), those who received consolidation with either melphalan alone or busulfan ($n$ = 14) and those who did not receive high-dose treatment for various reasons ($n$ = 48). Table 1 shows the demographic distribution of age, sex, stage, performance status, creatinine and $\beta 2$ microglobulin at presentation.

### Induction treatment

All 63 patients received courses of infusional chemotherapy, which included vincristine 0.4 mg i.v. by continuous infusion over 24 h for 4 days, doxorubicin 9 mg $m^{-2}$ i.v. by continuous infusion over 24 h for 4 days and methyl prednisolone 1.5 gm i.v. or p.o. for 4 days and then tapered and weekly cyclophosphamide (C-VAMP)

**Table 1** Patient demographics

| Patient characteristic | ABMT ($n$ = 26) | PBSCT ($n$ = 37) |
|---|---|---|
| Age (years) | | |
| < 50 | 12 (46) | 17 (46) |
| ≥ 50 | 14 (54) | 20 (54) |
| Median | 50 | 49 |
| Range | 40–67 | 37–63 |
| Sex | | |
| Male | 16 (62) | 23 (62) |
| Female | 10 (38) | 14 (38) |
| Stage | | |
| IA | 6 (23) | 5 (14) |
| IIA | 1 (4) | 4 (10) |
| IIIA | 14 (54) | 23 (62) |
| IIIB | 5 (19) | 5 (14) |
| Performance status | | |
| 0 | 20 (77) | 21 (57) |
| 1 | 6 (23) | 14 (38) |
| 2 | 0 (0) | 2 (5) |
| Serum creatinine ($\mu$mol $l^{-1}$) | | |
| < 130 | 25 (96) | 35 (95) |
| 130–200 | 1 (4) | 2 (5) |
| $\beta_2$-microglobulin (mg $l^{-1}$) | | |
| < 3 | 21 (81) | 24 (65) |
| ≥ 3 | 5 (19) | 13 (35) |

No significant difference by the Kruskal–Wallis test and chi-square test. Numbers in parentheses are percentages.

**Table 2** Response to C-VAMP induction treatment and after high-dose treatment

| Response | ABMT ($n$ = 26) | PBSCT ($n$ = 37) | $P$-value |
|---|---|---|---|
| *C-VAMP induction treatment* | | | |
| Complete remission | 7 (27) | 12 (32) | NS |
| Partial remission | 16 (61) | 17 (46) | NS |
| No response | 3 (12) | 8 (22) | NS |
| *After high-dose treatment* | | | |
| Complete remission | 22 (84) | 26 (70) | NS |
| Partial remission | 2 (8) | 10 (27) | NS |
| No response | 2 (8) | 1 (3) | NS |

NS, not significant. Numbers in parentheses are percentages.

**Table 3** Comparison of two harvest protocols used in PBSCT (protocol A and protocol B)

| | Protocol A ($n$ = 14) | Protocol B ($n$ = 23) | $P$-value |
|---|---|---|---|
| Mononuclear cells (× $10^8$ cells $kg^{-1}$) | 8.54 | 4.1 | 0.00 |
| Range | 2.26–11.91 | 2.37–9.14 | |
| CD 34+ cells (× $10^6$ cells $kg^{-1}$) | 5.65 | 0.5745 | 0.002 |
| Range | 0.05–31.6 | (0.002–6.96) | |
| GM-CFU (× $10^4$ cells $kg^{-1}$) | 6.62 | 3.085 | 0.04 |
| Range | 1.58–38.62 | 0.02–24.95 | |
| Platelet recovery (days)[a] | 18 | 19 | > 0.1 |
| Range | 13–40 | 12–148 | |
| Neutrophil recovery (days)[b] | 17 | 19 | > 0.1 |
| Range | 12–42 | 14–40 | |

[a]Platelet recovery to 50 × $10^9$ $l^{-1}$. [b]Neutrophil recovery to 0.5 × $10^9$ $l^{-1}$

as initial treatment following diagnosis (Raje et al, 1995). These courses were repeated every 21 days until plateau response occurred (see below) or the patients went into complete remission (CR). One further course was then given.

### High-dose treatment

Approximately 6 weeks after the start of the last chemotherapy cycle, high-dose treatment and an autograft with bone marrow or peripheral blood stem cells was given. Conditioning comprised melphalan (200 mg $m^{-2}$ infusion over 30 min) on day –1 if EDTA was > 40 ml $min^{-1}$. Adequate hydration before and after high-dose treatment with melphalan was ensured to maintain a urine output of 20 ml $min^{-1}$ 1 h after high dose and 500 ml $h^{-1}$ in the subsequent 2 h.

### ABMT

Between January 1989 and May 1992, 26 patients received an ABMT. Marrow was harvested as an inpatient under general anaesthesia, just before high-dose treatment. The target total nucleated cell collection was 2–5 × $10^8$ cells per kg of body weight. Marrow was stored following controlled cooling to – 140°C with 5% dimethylsulphoxide (DMSO) and stored in the vapour phase of liquid nitrogen. When required, it was rapidly thawed at 37°C and reinfused immediately without further processing.

### PBSCT

Between May 1992 and November 1995, 37 patients received PBSCTs using stem cells mobilized with recombinant human granulocyte colony-stimulating factor (rhG-CSF), all harvests being undertaken as a day case/outpatient procedure.

### Protocol A

Between May 1992 and June 1994 PBSC mobilization was achieved using home administration of subcutaneous rhG-CSF (Amgen) at a dose of 125 $\mu$g $m^{-2}$ 12 hourly for 7 days (14 doses) 6 weeks after the last chemotherapy cycle and just before the high-dose treatment. Leucapheresis was performed on 4 consecutive days, starting on day 5 (days 5–8) using the Cobe separator, and all cells collected were cryopreserved and reinfused after thawing. Fourteen patients received protocol A.

### Protocol B

From June 1994, G-CSF was given at a dose of 12–16 $\mu$g $kg^{-1}$ for 4 days, followed by leucapheresis on 2 consecutive days starting on day 4. Twenty-three patients received this protocol.

### Flow cytometry and GM-CSF assays

Aliquots of each PBSC harvest were counted and used for flow cytometric analysis within 2 h of collection. Mouse anti-human antibodies were used.

Light-density mononuclear cells were plated in soft agar at a concentration of 5 × $10^5$ per dish using a double-layer technique, with 100 $\mu$l of 5637 conditioned medium in the underlay as a source of GM-CSF. Cultures were plated in triplicate, incubated at 37°C in a humidified atmosphere and the colonies counted at 12–14 days using an inverted microscope.

© Cancer Research Campaign 1997

### Transplantation

Patients were admitted on the day they received the high-dose melphalan and transplants were usually undertaken in a four-bedded ward. This protocol had been approved by the hospital clinical research ethics committee and all patients signed an informed consent before recruitment. Prophylactic broad spectrum parenteral antibiotics were started routinely on the 5th day after transplant. Patients were discharged, if well, once the absolute neutrophil count reached $0.5 \times 10^9$ $l^{-1}$ and they no longer needed intravenous antibiotic support. No growth factors were used in the post transplant setting. The criteria for discharge were uniform throughout the programme.

### Maintenance interferon

After high-dose treatment, patients were started on maintenance interferon alpha (3 megaunits $m^{-2}$ 3 × weekly, S/C- Schering Plough) initially as part of a randomized trial (Cunningham et al, 1993; Powles et al, 1995) and subsequently in all patients when the WBC count reached $2 \times 10^9$ $l^{-1}$ and platelet count $50 \times 10^9$ $l^{-1}$. The dose of interferon was reduced or stopped according to haematological criteria or other toxic manifestations.

### Response and relapse criteria

Our response criteria and definition of CR have been described earlier (Gore et al, 1989). Four criteria had to be met for a patient to be regarded as having achieved CR: (1) no paraprotein measurable by scanning densitometry of serum proteins separated on cellulose acetate membrane by electrophoresis and stained with Ponceau S; (2) no detectable Bence-Jones proteinuria on electrophoresis of neat urine stained with colloidal gold; (3) 5% or fewer plasma cells of normal morphology on bone marrow aspiration; and (4) criteria 1–3 had to be fulfilled for at least 3 months. Patients were regarded as having achieved a partial response (PR) if there was a 50% decrease in measurable paraprotein (IgG or IgA myeloma) or bone marrow infiltration (non-secretory or Bence-Jones myeloma) that was sustained for a month or more. Relapse was defined as reappearance of paraprotein or bone marrow infiltration of more than 5% for patients in CR and as a 25% increase in measurable paraprotein in two samples 1 month apart for patients in PR. No response (NR) was considered if the patient failed to achieve a CR or a PR.

### Statistical considerations

Patient characteristics in the two arms were compared using the chi-square and Kruskal–Wallis test (Kruskal and Wallis, 1952). Engraftment data were plotted using Kaplan–Meier (Kaplan and Meier, 1958) life table curves, and comparisons were made using the log-rank test (Peto and Peto, 1972). The duration of response was measured from the date of start of induction treatment. Overall and progression-free survivals were plotted using the Kaplan-Meier method, and comparisons were again made using the log-rank method.

### RESULTS

Data have been analysed in April 1996 with a median follow-up of 30 months. The median follow-up in the PBSCT patients is 25

**Figure 1** Time to neutrophil recovery to $0.05 \times 10^9$ $l^{-1}$ after high-dose treatment. $P$ = NS

**Figure 2** Time to platelet recovery to $50 \times 10^9$ $l^{-1}$ after high-dose treatment. $P = 0.0015$

**Figure 3** Life table curves of in-hospital stay. $P = 0.0000$

months with the minimum follow-up being 6 months. Patient demography is described in Table 1, and the distribution of known prognostic criteria was the same for both groups.

### Induction treatment

Patients received induction with C-VAMP chemotherapy. The ABMT group of patients received a median of five courses (range 3–7), while the PBSCT group received a median of six courses of chemotherapy (range 1–8) before consolidation with high-dose treatment; this difference was not significant. Details of response to induction treatment are shown in Table 2 and are similar in the two arms, with an overall response before

© Cancer Research Campaign 1997

**Figure 4** Overall survival from start of induction treatment. $P$ = NS

**Figure 5** Progression-free survival from start of induction treatment. $P$ = NS

high-dose treatment of 88.4% in the ABMT and of 78.3% in the PBSCT patients ($P$ = NS).

### Autologous stem cell transplants

Twenty-six patients received an ABMT and are compared with 37 patients who received a PBSCT. The median total nucleated cell dose returned in the ABMT patients was $2.16 \times 10^8$ cells $kg^{-1}$ (range $1.11–2.26 \times 10^8$ cells $kg^{-1}$). The median cell dose returned in the PBSCT patients was $5.1 \times 10^8$ cells $kg^{-1}$ (range $2.26–11.91 \times 10^8$ cells $kg^{-1}$). Details of response following high-dose treatment are shown in Table 2 and are similar in the two arms.

For PBSCT harvests, 14 patients underwent mobilization with protocol A and 23 patients with protocol B. Details of the quality of their harvests are shown in Table 3. Even though both CD 34+ numbers and the GM-CFUs were significantly different in the two groups, engraftment was no different. Neutrophil recovery to $0.5 \times 10^9$ $l^{-1}$ occurred at a median of 17 days (range 12–42 days) in protocol A vs 19 days (range 14–40 days) in protocol B ($P$ = NS). Platelet recovery to $50 \times 10^9$ $l^{-1}$ was also similar (median 18 days, range 13–40 days vs median 19 days, range 12–148 days; $P$ = NS). There was no difference with respect to survival outcome and relapse in these two groups and therefore subsequent analysis has been undertaken with the two groups combined.

### Engraftment and days in hospital

Figures 1 and 2 show the time to neutrophil and platelet recovery respectively. A median of 22 days (range 12–38 days) was required to reach a neutrophil count of $0.5 \times 10^9$ $l^{-1}$ in the ABMT group compared with 19 days (range 12–42 days) in the PBSCT group ($P$ = NS). Platelet recovery to $50 \times 10^9$ $l^{-1}$ was significantly faster in the PBSCT patients with medians reached at 19 days (range 12–148 days) compared with 33 days (range 18–72 days) in the ABMT patients ($P$ = 0.0015).

The ABMT patients spent a median of 27 days in hospital, while the PBSCT patients spent a median of 20 days from transplant ($P$ = 0.00001) (Figure 3).

**Figure 6** Probability of starting interferon after high-dose treatment. $P$ = NS

### Survival and relapse

Figures 4 and 5 show the overall survival (OS) and progression-free survival (PFS) in the two groups of patients. The median OS and PFS for the PBSCT has not yet been reached. Of the ABMT patients, 76.9% (95% CI 60–93%) compared with 85.3% (95% CI 72–99%) of the PBSCT patients are alive at 3 years ($P$ = NS). Of the ABMT patients, 53.8% (95% CI 34–73%) do not have evidence of disease progression at 3 years compared with 57.6% (95% CI 34–81%) of the PBSCT patients ($P$ = NS). The probability of relapse at 3 years was 42.3% in the ABMT arm vs 40% in the PBSCT arm ($P$ = NS). Within the PBSCT arm, there was no difference in the OS and PFS between patients receiving either protocol A or protocol B.

### Interferon maintenance

Interferon maintenance was started at a median of 58 days (range 33–146 days) in the ABMT patients and a median of 55 days (range 20–181 days) in the PBSCT patients ($P$ = NS) (Figure 6) and was continued until relapse. Tolerance to interferon was similar in the two groups. The commonest side-effect was flu-like symptoms and was seen in 60% of the patients in both arms. Other side-effects, such as headache (one patient in each arm), skin problems (one patient in ABMT arm and two patients in PBSCT arm), diarrhoea (one patient in each arm) and sexual problems (three patients in the ABMT arm and one patient in the PBSCT arm) were rarely seen. Two patients in the ABMT arm discontinued interferon treatment, one because of depression and the other because of petit mal seizures. Similarly, two patients belonging to the PBSCT group also discontinued treatment, the reasons being depression and psoriasis. Dose modification was seen in 11 ABMT and 13 PBSCT patients.

© Cancer Research Campaign 1997

### DISCUSSION

High-dose alkylating chemotherapy with haemopoietic rescue is being used more frequently as consolidation therapy for myeloma, and blood-derived stem cells (Fermand et al; 1993; Attal et al, 1995; Harousseau et al, 1995*a*; Powles et al, 1995) have become increasingly the method used, but as yet the significance of tumour cell contamination in PBSCT is unknown.

The French Registry has previously reported on a retrospective comparison of ABMT and PBSCT (Harrousseau et al, 1995*b*). These comparisons were made in patients belonging to 18 French centres with significant demographic differences in the two groups with respect to patient age and chemotherapy before transplant, giving a better prognostic bias to the PBSCT cohort. No significant difference was noted by them with respect to response and survival outcome.

We have compared the two procedures at a single centre with no difference in the prognostic variables in the two groups. All patients have received identical induction treatment and conditioning regimens. The only difference in the two groups was the source of the stem cells. Our data show a more rapid platelet recovery in the PBSCT group than in the ABMT group (19 days vs 33 days), but neutrophil recovery was not significantly different (19 days vs 22 days). However, our PBSCT patients had a significantly shorter hospital stay. This was not because of any change in our discharge criteria and it remains unclear why our PBSC patients left hospital earlier. Comparison of our results with the French data (Harousseau et al, 1995*b*) shows some important differences. They show a more rapid neutrophil recovery in the PBSCT arm (13 days vs 22 days; $P < 0.001$) and no significant difference in platelet recovery (26 days in PBSCT patients vs 22 days in ABMT patients). This could be attributed to differences in mobilization schedules used. We have used growth factors alone for mobilization, whereas the French study had harvested PBSC after chemo-induced aplasia and without priming with haematopoietic growth factors. Our results also show a delay of about 5–8 days with respect to neutrophil recovery in our PBSCT group compared with other series (Fermand et al, 1993; Harousseau et al, 1995*b*). This could well be because of the low numbers of CD34+ cells and GM-CFU counts in our PBSCT harvests and highlights the fact that mononuclear counts are not good indicators of the quality of graft.

Our CR rate was not significantly different in the two arms (70% vs 84%). The lower CR rate in the PBSCT arm may be the result of a shorter follow-up in the PBSCT patients, because it can take as long as 786 days (Singhal et al, 1995) for paraprotein to disappear following high-dose treatment. Our minimum follow-up in the blood stem cell patients is 6 months, and there is the theoretical possibility of seeing more patients achieving CR beyond 6 months because of the biology of the disease.

Reinfusion of the malignant clone is a major concern in all autografting procedures. Corradini et al (1995) have demonstrated, by a polymerase chain reaction (PCR)-based strategy using clone-specific sequences derived from the rearrangement of IgH genes, the presence of both pre-and post-switch B cells in bone marrow and blood stem cell harvests. Whether or not these cells contribute to relapse is debatable. Their data suggest that blood-derived stem cell harvests may have a lower rate of contamination.

Limited data are available on the optimal dose and quality of PBSC harvests required for adequate engraftment and, although various investigators have identified favourable predictors of engraftment (Tricot et al, 1995; Demirer et al, 1996), the minimum dose required for engraftment is unknown. However it seems logical that a lower dose of haemopoietic stem cells will relate to a lower reseeding with malignant cells. A dose of $5 \times 10^6$ CD34+ cells $kg^{-1}$ has been identified as adequate (Bensinger et al, 1994) for rapid engraftment, but we have seen sustained engraftment with significantly lower doses of CD 34+ cells. Newer approaches such as positive selection of stem cell harvests are being applied (Gazitt et al, 1995; Schiller et al, 1995) and a comparison of CD34+ selected vs unselected PBSCT will be crucial in identifying a superior quality graft in myeloma. Retroviral marking of CD34-enriched harvests has been undertaken in myeloma (Dunbar et al, 1995) and will undoubtedly be an area for future therapeutic trials.

The OS and PFS in our patients compare favourably with those reported by other investigators. The University of Arkansas (Barlogie et al, 1995) have reported a median OS of 40 months and a median event-free survival of 23 months in 287 patients who have undergone autotransplants. The French registry has reported a median OS of 54 months in responding patients with a median remission duration of 33 months (Harousseau et al, 1995*a*). Fermand et al (1993) have reported a median OS of 59 months and a median event-free survival of 43 months. We were unable to see a significant survival disadvantage from the use of G-CSF-generated stem cells, obviating the concern of mobilization and reseeding by the PBSCT of tumour cells.

Interferon maintenance following high-dose treatment has shown benefit (Cunningham et al, 1993; Powles et al, 1995) to patients after autografting, and it is important that the PBSC graft should tolerate its use. We have previously documented the robustness of peripheral blood stem cells to interferon therapy (Powles et al, 1996), and this study confirms that interferon could be started on all patients receiving a PBSCT and that it was as well tolerated in this group as in the ABMT group.

What happens to the patient after autografting is dictated by the remaining disease cells in the host and slight variations in transplant procedures, i.e. ABMT or PBSCT, which even though capable of influencing engraftment, may not influence the ultimate outcome. All autograft strategies will, however, be effective only if it is possible to further escalate high-dose regimens to eliminate residual disease.

### ACKNOWLEDGEMENTS

The authors would like to acknowledge the Bud Flanagan Leukaemia Fund, the Cancer Research campaign and the Institute of Cancer Research.

### REFERENCES

Attal M, Harousseau JL, Stoppa AM, Sotto JJ, Fuzibet G, Rossi JF, Casassus P, Thyss A, Maisonneuve H, Facon T, Ifrah N, Payen C and Bataille R (1995) High dose therapy in multiple myeloma: final analysis of a prospective randomized study of the "Intergroupe Francais Du Myelome". *Blood* **86**: 124a

Barlogie B, Hall R, Zander A, Dicke K and Alexanian R (1986) High-dose melphalan with autologous bone marrow transplantation for multiple myeloma. *Blood* **67**: 1298–1301

Barlogie B, Anderson K, Berenson J, Crowley J, Cunningham D, Gertz M, Henon P, Horowitz M, Jagannath S, Powles R, Reece D, Reiffers J, Salmon S, Tricot G and Vesole D (1995) Transplants for multiple myeloma. *Bone Marrow Transplant* **15**: s234–239

Belch AR, Szczepek A, Bergsagel PL and Pilarski LM (1995) Circulating CD34+ cells from peripheral blood in multiple myeloma include B cells with patient specific IGH CDR3 sequences and CD34mRNA as well as DNA hyperdiploidy and N-ras mutation. *Blood* **86**: 276a

© Cancer Research Campaign 1997

Bensinger WI, Longin K, Appelbaum F, Rowley S, Weaver C, Lilleby K, Gooley T, Lynch M, Higano T, Klarnet J, Chauncey T, Storb R and Buckner CD (1994) Peripheral blood stem cells (PBSCs) collected after recombinant granulocyte colony stimulating factor (rhG-CSF): an analysis of factors correlating with the tempo of engraftment after transplantation. *Br J Haematol* **87**: 825–831

Corradini P, Voena C, Astolfi M, Ladetto M, Tarella C, Boccadoro M and Pileri A (1995) High-dose sequential chemoradiotherapy in multiple myeloma: residual tumour cells are detectable in bone marrow and peripheral blood stem cell harvests and after autografting. *Blood* **85**: 1596–1602

Cunningham D, Powles R, Malpas J, Milan S, Meldrum M, Voner C, Montes A, Hickish T, Nicolson M, Johnson P, Mansi J, Treleaven J, Raymond J and Gore ME (1993) A randomised trial of maintenance therapy with Intron-A following high dose melphalan and ABMT in myeloma. *ASCO Abstracts* **12**: 364

Cunningham D, Paz-Ares L, Milan S, Powles R, Nicolson M, Hickish T, Selby P, Treleaven J, Viner C, Malpas J, Slevin M, Findlay M, Raymond J and Gore ME (1994) High dose melphalan and autologous bone marrow transplantation as consolidation in previously untreated myeloma. *J Clin Oncol* **12**: 759–763

Demirer T, Buckner CD, Gooley T, Appelbaum FR, Rowley S, Chauncey T, Lilleby K, Storb R and Bensinger WI (1996) Factors influencing collection of peripheral blood stem cells in patients with multiple myeloma. *Bone Marrow Transplant* **17**: 937–941

Dimopoulos MA, Alexanian R, Przepiorka D, Hester J, Andersson B, Giralt S, Mehra R, Besien K, Delasalle K, Reading C, Deisseroth A and Champlin R (1993) Thiotepa, Busulfan and Cyclophosphamide: a new preparative regimen for autologous marrow or blood stem cell transplantation in high risk myeloma. *Blood* **82**: 2324–2328

Dunbar CE, Cottler-Fox M, O'Shaoghnessy JA, et al (1995) Retrovirally marked CD34-enriched peripheral blood and bone marrow cells contribute to long term engraftment after autologous transplantation. *Blood* 3048–3057

Fermand JP, Chevret S, Ravaud P, Divine M, Leblond V, Dreyfus F, Mariette X and Brouet J (1993) High-dose chemoradiotherapy and autologous blood stem cell transplantation in multiple myeloma: results of a phase II trial involving 63 patients. *Blood* **82**: 2005–2009

Gazitt Y, Reading C, Hoffman R, Wickrema A, Vesole D, Jagannath S, Condino J, Lee B, Barlogie B and Tricot G (1995) Purified CD34+ Lin– Thy+ stem cells do not contain clonal myeloma cells. *Blood* **86**: 381–389

Gore ME, Selby PJ, Viner C, Clark PI, Meldrum M, Millar B, Bell J, Maitland JA, Milan S, Judson IR, Zuiable A, Tillyer C, Slevin M, Malpas JS and McElwain TJ (1989) Intensive treatment for multiple myeloma and criteria for complete remission. *Lancet* **11**: 879–882

Harousseau JL, Attal M, Divine M, Marit G, Leblond V, Stoppa A, Bourhis J, Cailot D, Boassan M, Abgrall J, Facon T, Linassier C, Cahn Y, Lamy T, Troussard X, Gratecos N, Pignon B, Auzanneau G and Bataille R (1995*a*) Autologous stem cell transplantation after first remission induction treatment in multiple myeloma: a report of the French Registry on autologous transplantation in multiple myeloma. *Blood* **85**: 3077–3085

Harousseau JL, Attal M, Divine M, Milpied N, Marit G, Leblond V, Stoppa A, Bourhis J, Caillot D, Boasson M, Agrall J, Facon T, Colombat P, Cahn J, Lamy T, Troussard X, Gratecos N, Pignon B and Auzanneau G (1995*b*) Comparison of autologous bone marrow transplantation and peripheral blood stem cell transplantation after first remission induction treatment in multiple myeloma. *Bone Marrow Transplant* **15**: 963–969

Kaplan EL and Meier P (1958) Non-parametric estimation from incomplete observations. *J Am Stat Assoc* **53**: 457–481

Kruskal WH and Wallis WA (1952) Use of ranks in one-criterion variance analysis. *J Am Stat Assoc* **47**: 583–621

Mariette X, Fermand JP and Brouet JC (1994) Myeloma cell contamination of peripheral blood stem cell grafts in patients with multiple myeloma treated by high-dose therapy. *Bone Marrow Transplant* **14**: 47–50

McElwain TJ and Powles RL (1983) High dose intravenous melphalan for plasma cell leukemia and myeloma. *Lancet* **2**: 822–824

Peto R and Peto J (1972) Asymptomatically efficient invariant procedures. *J R Stat Soc A* **135**: 185–206

Powles RL, Raje NS, Cunningham D, Malpas J, Milan S, Horton C, J Mehta, S Singhal, C Viner and J Treleaven (1995) Maintenance therapy for remission in myeloma with Intron A following either an autologous bone marrow transplantation or peripheral stem cell rescue. *Stem Cells* **13**: 114–117

Powles R, Raje N, Horton C, Mehta J, Singhal S, Hickish T, Viner C, Milan S, Treleaven J and Cunningham D (1996) Comparison of interferon tolerance after autologous bone marrow or peripheral blood stem cell transplants for myeloma patients who have responded to induction therapy. *Leukaemia and Lymphoma* **21**: 421–427

Raje NS, Powles RL, Horton CAP, et al (1995) Peripheral blood stem cell transplantation in multiple myeloma. *Br J Haematol* **89** (s1): 84

Rubia J, Bonanad S, Palau J, Sanz G and Sanz M (1994) Rapid progression of multiple myeloma following G-CSF mobilization. *Bone Marrow Transplant* **14**: 475–476

Schiller G, Vescio R, Freytus C, Spitzer G, Sahebi F, Lee M, Wu C, Cao J, Lee J, Hong C, Lichtenstein A, Lill M, Hall J, Berenson R and Berenson J (1995) Transplantation of CD 34+ peripheral blood progenitor cells after high dose chemotherapy for patients with advanced multiple myeloma. *Blood* **86**: 390–397

Selby PJ, McElwain TJ, Nandi AC, Perren TJ, Powles RL, Tillyer CR, Osborne RJ, Slevin ML, and Malpas JS (1987) Multiple myeloma treated with high dose intravenous melphalan. *Br J Haematol* **66**: 52–55

Singhal S, Powles R, Milan S, Raje N, Viner C, Treleaven J, Raymond J, Cunningham D and Mehta J (1995) Kinetics of paraprotein clearance after autografting for multiple myeloma. *Bone Marrow Transplant* **16**: 537–540

Tricot G, Jaganath S, Vesole D, Nelson J, Tindle S, Miller L, Cheson B, Crowley J and Barlogie B (1995) Peripheral blood stem cell transplantation for multiple myeloma: identification of favourable variables for rapid engraftment in 225 patients. *Blood* **85**: 588–596

© Cancer Research Campaign 1997